# A scalable school‐based intervention to increase early adolescents' motor competence and health‐related fitness

**DOI:** 10.1111/sms.14410

**Published:** 2023-05-25

**Authors:** Mikko Huhtiniemi, Arja Sääkslahti, Asko Tolvanen, David R. Lubans, Timo Jaakkola

**Affiliations:** ^1^ Faculty of Sport and Health Sciences University of Jyväskylä Jyväskylä Finland; ^2^ Faculty of Education and Psychology University of Jyväskylä Jyväskylä Finland; ^3^ Centre for Active Living and Learning, School of Education The University of Newcastle Callaghan New South Wales Australia

**Keywords:** adolescents, fitness, intervention, motor competence, school

## Abstract

Schools are key settings for the promotion of students' physical activity, fitness, and motor competence. The purpose of our study was to investigate the efficacy of a 5‐month‐long intervention program that aimed to increase students' motor competence and health‐related fitness during school days. We conducted a quasi‐experimental study with 325 Finnish Grade 5 (*M*_age_ = 11.26, SD = 0.33) students from five schools. Two schools were allocated to the intervention group and three schools to the control group. The intervention consisted of three components: (a) weekly 20 min session during regular PE lessons, (b) weekly 20 min session during recess, and (c) daily 5‐minute‐long classroom activity breaks. All activities were designed to systematically develop different elements of motor competence and fitness. The following assessments were conducted at baseline and 5‐months: cardiorespiratory fitness levels were measured by 20‐meter shuttle run test, muscular fitness by curl‐up and push‐up tests, and motor competence by 5‐leaps and throwing–catching combination tests. We analyzed the data using a multi‐group latent change score modeling. Results showed that students in the intervention group developed significantly better in 20‐meter shuttle run test (*β* = 0.269, *p* = 0.000, 95% CI [0.141, 0.397]; +5.0 laps), push‐up (*β* = 0.442, *p* = 0.000, 95% CI [0.267, 0.617]; +6.5 repetitions), curl‐up (*β* = 0.353, *p* = 0.001, 95% CI [0.154, 0.552]; +7.8 repetitions), and throwing–catching combination tests (*β* = 0.195, *p* = 0.019, 95% CI [0.033, 0.356]; +1.1 repetitions) than students in the control group. The intervention program appeared to be feasible and effective in increasing students' cardiorespiratory fitness, muscular fitness, and object control skills. This indicates that guided school‐based physical activity programs can be influential in promoting physical fitness and motor competence among early adolescent students.

## 1 INTRODUCTION

It is evident that a vast majority of children and adolescents do not meet the current physical activity guidelines.^1^ Concurrently, negative trends have been documented in young people's motor competence^2^ and health‐related fitness.^3^, ^4^ These findings are alarming as lowered physical performance in adolescence has been shown to negatively influence several health outcomes such as weight status and cardiovascular disease risk factors.^5^, ^6^, ^7^, ^8^ Drawing from these findings, a wide range of actions in multiple domains have been explored to reverse the negative course. The school setting has been identified as one of the most compelling contexts to promote motor competence and health‐related fitness as it effectively reaches the whole age cohort of children and adolescence.^9^, ^10^ In addition to the effective reach of the population, schools also have teachers and other experts with access to equipment and facilities for physical activity promotion.^11^ Therefore, the aim of this study was to investigate the effectiveness of a multicomponent school‐based intervention that targeted early adolescent students' motor competence and health‐related fitness.

Motor competence is a global term to describe goal‐directed human movement and is often used interchangeably with the following: motor proficiency, motor ability, and motor coordination.^12^ It has been shown that for optimal motor competence development during childhood systematic training and practice are needed.^13^, ^14^, ^15^ The proficient level and progression of motor competence form the building blocks for various physical activities, sport skills, and motor behaviors across the life course.^13^, ^16^ Previous studies and systematic reviews have shown that motor competence is associated with several important health‐related factors, including higher levels of CRF and muscular fitness,^14^, ^17^, ^18^, ^19^ higher physical activity engagement in organized settings,^20^ and improved weight status.^5^, ^17^ Moreover, previous studies have demonstrated the potential of school‐based interventions to improve students' motor competence. For example, in a meta‐analysis including 56 trials and over 48 000 participants (aged 3–18 years) quality‐based PE interventions were positively related with increases in motor competence (pooled effect size: Hedges *g* = 0.38; 95% CI = 0.27–0.49).^9^ Another review and meta‐analyses by Dudley et al. showed that the highest effects in PE learning interventions were observed in psychomotor outcomes (e.g., motor competence, fundamental movement skills) (*d* = 0.52) followed by affective (*d* = 0.47), social (*d* = 0.32), and cognitive (*d* = 0.17) outcomes.^21^ Moreover, the systematic review and meta‐analysis by Morgan et al. found that school‐based programs delivered by physical education (PE) professionals can improve children's motor competence (standardized mean difference [SMD] = 1.42, 95% CI = 0.68–2.16).^22^

Health‐related fitness (HRF) is a multidimensional construct comprising cardiorespiratory fitness (CRF), musculoskeletal fitness, flexibility, and body composition.^7^, ^23^ Previous research has well established that HRF in youth, especially cardiorespiratory and muscular fitness, are significant markers of overall health.^7^, ^8^, ^24^, ^25^ Moreover, previous studies have demonstrated that school‐based interventions can improve young people's HRF. For example, Villa‐Gonzales et al. concluded that school‐based activities that include strength exercises may enhance muscular fitness among primary school students (aged <13 years).^26^ In another review and meta‐analytic study, school‐based interventions which targeted muscular fitness in adolescent boys showed small to‐moderate effects.^27^ Moreover, Hartwig et al. showed that physical activity interventions have a modest effect on CRF among 4–18‐year‐old students based on a pooled analysis of 20 controlled trials (overall effect: 0.47 mL/kg/ min [95% CI 0.33 to 0.61]).^28^

Although there is a wealth of information regarding the effects of different physical activity interventions on young people's motor competence^22^, ^29^, ^30^, ^31^ and health‐related fitness,^10^, ^26^, ^27^, ^28^ more studies especially in the school context are needed. In a recent Delphi study utilizing 46 experts in the field, school‐based interventions for increasing HRF that are feasible and scalable was ranked fourth in international priorities for physical fitness research.^32^ Hence, more evidence‐based empirical procedures to contribute students' motor competence and health‐related fitness are needed. Additionally, there is a need for programs where the cost‐effectiveness and the potential scalability to wider use have been considered.^32^ This intervention has the potential to be scaled‐up to improve public health among Finnish students. As noted by Milat and colleagues for interventions to be scalable, they need to have: (i) evidence of effectiveness, (ii) potential for extended reach, (iii) show high acceptability among the target population and setting, and (iv) acceptable delivery costs.^33^, ^34^ Interventions that are integrated into existing school structures such as PE lessons and recess time are more likely to be scalable because they address the criteria outlined above.^35^ Stemming from these arguments, our study adds to the existing literature by examining the effects of an intervention program that was integrated into PE lessons, academic lessons, and recess time.

More specifically, the aim of our study was to assess the effectiveness of a multicomponent school‐based intervention on adolescents' motor competence and HRF. We hypothesized that students allocated to the intervention group would have significantly higher CRF, muscular fitness, locomotor, and object control skill proficiency compared to students in the control group. Additionally, the effects of body mass index (BMI) and gender were investigated in our analysis.

## 2 METHOD

### 2.1 Participants and recruitment

The reporting of the current study was aligned with the Transparent Evaluations with Non‐Randomized Design (TREND) statement.^36^ A quasi‐experimental intervention design, with experimental and control groups, and pre‐ and posttests was implemented. Participants of the study were 325 Finnish Grade 5 students (baseline *M*_age_ = 11.26, SD = 0.33) from Central‐Finland. Participants represented 16 classes from five schools that were conveniently selected based on their distance from the University. In the beginning of the study, schools were allocated to intervention or control groups. The intervention group consisted of 157 students (78 boys and 78 girls, one student unknown) from two schools and seven classes. The control group consisted of 168 students (81 boys and 83 girls, four students unknown) from three schools and nine classes. The study schools represented typical Finnish elementary schools, and they shared many attributes that were similar. All schools were similar in size, had similar indoor and outdoor facilities for physical activity, had teachers with similar master‐level education and teaching experience (general classroom teachers), and had similar student populations (e.g., ethnicity mostly white, drawing from similar neighborhoods). Both control and intervention schools followed the same national core curriculum for PE which specifies the overall aims in PE and guides the selection of different contents and methods.^37^, ^38^

The students' guardians were informed about the study protocols and provided written informed consent for their children to participate in the study. The study protocol was approved by the ethics committee of the local university.

### 2.2 Intervention description and components

The intervention program aimed to increase students' motor competence and health‐related fitness through weekly guided activities, aligned with the aims of the national PE curriculum. The 5‐month‐long program was implemented during regular school days and consisted of the three components described below. Two example weeks elaborating the intervention activities and structure in more detail are provided in the Supporting Information Table S1. The selection and design of the components were aligned with the Theory of Expanded, Extended, and Enhanced Opportunities (TEO) which describes a practical threefold taxonomy to identify intervention targets and to better understand physical activity engagement of youth.^35^ The three elements of TEO include: (a) the expansion of opportunities to be active (e.g., breaks during academic lessons), (b) the extension of an existing physical activity opportunity (e.g., a long recess devoted for activity), and (c) the enhancement of existing physical activity opportunities (e.g., increasing the intensity of PE lessons).

#### 2.2.1 PE lesson component

A 20‐minutes‐long specific fitness and motor competence unit that was implemented weekly in the beginning of students' regular 90‐minutes physical education lesson (i.e., extended warm‐up). The unit contained different activities, games, and tasks that systematically developed different elements of physical fitness and motor competence. Each unit targeted one or two different elements, such as CRF and throwing skills through a certain game or movement task (see Supporting Information Table S1 for detailed examples). This component was delivered by two trained researchers.

#### 2.2.2 Recess component

A 20 min‐long recess activity that was implemented once a week during students' regular recess time. Similar, to the PE lesson component, the recess component contained a variety of activities, games, and tasks that systematically developed different elements of fitness and motor competence (see Supporting Information Table S1 for detailed examples). To effectively use the recess time, students were instructed for the activities at the end of the preceding academic lesson. This component was delivered by two trained researchers.

#### 2.2.3 Classroom activity breaks

Daily 5‐minute‐long activity breaks were organized during academic lessons. The research team designed the activities and their order of implementation. Each activity break was designed to promote a certain physical performance indicator, for example muscular fitness (see Supporting Information Table S1 for detailed examples). The breaks were administered by the class teachers who followed written instructions and education given by the research team.

All activities and their implementation were planned by the research team. All classes in the intervention group received the same standardized program. The total duration of the intervention program was 5 months starting in October and finishing in the end of March. During regular school breaks (e.g., Christmas holiday period) there were no activities, and therefore, the program consisted of 18 active weeks with 65 min of structured content per week. The weekly activities were instructed by trained research assistants who were fifth‐year master's degree students from the PE teaching education program of the local university. The various games, tasks and other activities chosen to the program were easy to implement and did not require any special equipment or other special arrangements. Tasks and activities included an abundance of variation (e.g., easy or hard option for a certain task) to enable students with different fitness and skill levels to participate. Moreover, students' engagement and commitment were fortified by including a student‐designed elements to the intervention program. Each class designed their own favorite activity week which was then executed at the end of the intervention period.

Students in the control group did not receive the intervention but they had a similar school week structure. Physical education classes (one 90 min class once a week) and recesses (one long recess per day but without guided activities) were structured in a similar way compared to the intervention schools. After the post‐measurements, the intervention activities were also provided to the control group schools.

### 2.3 Intervention fidelity and adherence

We confirmed intervention fidelity through a set of measures. A predesigned structured intervention plan was followed in all intervention classes that allowed the research team to evaluate how precisely the plan was executed. Research assistants used lesson plans to deliver the activities and they were instructed to write down all possible changes at the end of each lesson. They were also instructed to report possible problems regarding participation or other influencing factors. In addition, the regular teacher of the class was present during the activities and was also guided to report any issues (e.g., misbehaviors). The research team met weekly to discuss the implementation process. Teachers or research assistants did not report any deviations from the structured plan or other problems during the intervention period.

Participation rate was monitored throughout the study. As the intervention program was integrated to the school day structure, all students in the class took part in the activities, including those who did not want to participate in the study. During the intervention period, regular absences were reported resulting typically from short‐term sickness. All intervention group students participated to at least 16 out of 18 intervention weeks which was considered acceptable, and therefore, no students were excluded from the analysis.

### 2.4 Measures

Baseline measures were conducted in September 2018 for both groups. The intervention program started 2 weeks after the pretests. The weekly program followed the regular yearly schedule of Finnish schools. All posttests were completed in April 2019 for both groups. The intervention group completed posttests 1 week after the end of the program.

All physical performance tests were administered by the trained researchers for both intervention and control groups in pre‐ and post‐testing phases. Researchers who conducted the measurements were blinded regarding the groups condition. All tests were completed in gym hall during a 90‐minute class, and the order of tests was the following: (1) throwing–catching combination test, (2) 5‐leaps test, (3) curl‐up test, (4) push‐up test, and (5) 20‐meter shuttle run test. In addition, students' height and weight were measured. A separate session was organized to collect students' background information using a questionnaire administered during a regular school class.

#### 2.4.1 Motor competence

Students' motor competence was measured using two product‐oriented measures; the throwing–catching combination test (object control skills) and 5‐leaps test (locomotor skills).^39^ In the throwing–catching combination test, the participant throws a tennis ball at a target area 1.5 × 1.5 m square situated at 90 cm above floor level. Throwing distance is 7 m for girls and 8 m for boys. The participant throws the ball behind a marked line, hits the target area, and catches the ball after one bounce. The final score is the number of correctly performed throwing–catching combinations from 20 attempts. In the 5‐leaps test, the participant completes five leaps, beginning and finishing with the legs in a parallel position. The final test score was the distance from the start to finish position measured from the heel of the nearest foot. Both motor competence tests have demonstrated acceptable validity and reliability among Finnish adolescents.^40^, ^41^ More specifically, the test–retest intraclass correlations have been adequate in both throw‐catch combination (0.69) and 5‐leaps (0.84) tests.^39^

#### 2.4.2 Cardiorespiratory fitness

Students' CRF was measured using the 20‐meter shuttle run test also referred as the Progressive Aerobic Cardiovascular Endurance Run (PACER).^42^ In the test, the participant runs continuously between two lines that are set 20 m apart following a progressive cadence. The final score is the number of shuttles reached before the participant is unable to keep pace with the signals.

#### 2.4.3 Muscular fitness

Students' muscular fitness was measured with curl‐up and push‐up tests.^39^ In the curl‐up test, participants lie on their back with legs bent to 100 degrees and curl‐up slowly keeping their heels on the floor. A measuring strip is located on the mat under their legs such that, in the start position, their fingertips are resting on the nearest edge of the measuring strip. As the participants curl‐up, their fingers slide across the strip. When their fingertips reach the other side of the measuring strip, the participants curl back down until their head touches the floor. Before the actual test, a participant was allowed tries. The test score is the number of correctly completed curl‐ups performed before being unable to keep pace with the rhythm set by the audio recording or when they reach 75 curl‐ups. The push‐up test is conducted for boys and the girls in a slightly different way. Boys use a starting position where their hands and toes touch the floor, whereas girls use a starting position where their hands and knees touch the floor. In both versions of the test, body and legs are kept in a straight line and arms are shoulder‐width apart. In the test, a participant lowers their body until there is a 90‐degree angle in the elbows (with the upper arms parallel to the floor). The final score is the number of correctly completed push‐ups in 60 seconds. Both muscular fitness tests have shown adequate reliability and validity among Finnish adolescents.^39^

#### 2.4.4 Body mass index

Participants' weight and height were measured using a calibrated scale and a portable measuring equipment to the nearest 0.1 kg and 0.1 cm, respectively. Participant were barefoot and wearing light clothes. Participants body mass index was calculated using a weight (kg) and height (m) formula (kg/m^2^).^43^

### 2.5 Statistical analyses

Preliminary analyses were performed with IBM SPSS 26 software.^44^ Data were first inspected for inputting errors, outliers, and missing data patterns. Following this, descriptive statistics and standardized data values were calculated. Because fitness measurements procedures were different between boys and girls in push‐ups and throwing–catching combination tests, the standardization was performed separately for boys and girls using pretest means and variances. Main analyses were conducted using Mplus 8.6 software.^45^ Latent change score analysis, in the structural equation modeling framework, was used to examine the effects of the intervention in the 20‐meter shuttle run, 5‐leaps, curl‐up, push‐up and throwing–catching combination tests, along with the body mass index. Baseline scores were controlled in the analysis. All analyses followed the intention‐to‐treat principles.^46^

Latent change score modeling combines features from cross‐lagged regression modeling and latent growth curves.^47^, ^48^, ^49^ In latent change models, the change between T0 and T1 is represented as a latent variable with a mean (i.e., average change), a variance (i.e., individual differences in change), and a covariation of the change with the initial factor and possible other factors in the model.^49^ This means that the model can estimate latent means and latent intraindividual mean changes (e.g., between pretest and posttest) but also interindividual differences in these variables.^50^ The latent change score modeling has been successfully applied in previous intervention studies.^50^, ^51^, ^52^

In the current study, a multi‐group latent change modeling was used to test the differences between groups. More specifically, mean parameters for latent change were constrained to be equal between boys and girls, and between intervention and control groups, and the subsequent change in model fit was evaluated. Wald test was used to test the difference between intervention and control groups, and between boys and girls.^45^

To take possible non‐normality of data into account, the robust full information maximum likelihood estimator (MLR) was used in the analyses. The standard procedure for handling missing values in Mplus was used, which utilizes all observations in the data without imputing the data.^45^ Multiple indicators were used to evaluate the overall model fit. More specifically, the chi‐square goodness‐of‐fit statistics (χ^2^), the comparative fit index (CFI), the Tucker‐Lewis index (TLI), root mean square error of approximation (RMSEA), and standardized root mean square residual (SRMR) were used. Following guidelines by Hu and Bentler, the model fit was considered good when values of CFI and TLI are close to 0.95, the SRMR is lower than 0.08, and the RMSEA is lower than 0.06.^53^

## 3 RESULTS

### 3.1 Preliminary analyses

Graphical inspection showed that the variables were approximately normally distributed. Values for skewness and kurtosis were below 1.2. In addition, no significant outliers were detected. The missing completely at random (MCAR) test (χ^2^ = 210.8, *df* = 186, *p* = 0.103) demonstrated that the data with and without missing values were similar, and thus, the missing data were considered to be missing completely at random.^54^

The means and standard deviations for both intervention and control groups are presented in Table 1. All bivariate correlations among study variables are presented in Supporting Information Table S2.

**TABLE 1:** Means and standard deviations for control and intervention groups at pre‐ and posttests.

	Control group		Intervention group	
	Pretest	Posttest	Pretest	Posttest
	*M* (SD)	*M* (SD)	*M* (SD)	*M* (SD)
*All*				
Body mass index (kg/m^2^)	18.66 (2.82)	18.99 (2.94)	18.46 (2.48)	18.97 (2.57)
20‐m shuttle run test	38.71 (17.81)	42.06 (18.22)	36.66 (18.56)	45.06 (19.33)
5‐leaps	7.91 (0.97)	8.17 (1.04)	8.11 (0.87)	8.38 (0.90)
Catching–throwing	11.61 (4.67)	12.91 (4.69)	11.25 (5.36)	13.65 (4.74)
Curl‐up	45.39 (23.29)	42.47 (21.00)	37.59 (21.79)	42.49 (21.59)
Push‐up	24.14 (13.31)	23.15 (13.38)	20.66 (11.99)	26.16 (14.34)
*Boys*				
Body mass index (kg/m^2^)	18.55 (2.69)	19.12 (3.07)	18.61 (2.64)	19.21 (2.77)
20‐m shuttle run test	42.44 (18.70)	45.45 (19.21)	40.22 (21.27)	48.63 (22.89)
5‐leaps	7.92 (0.94)	8.16 (1.10)	8.15 (0.89)	8.40 (0.89)
Catching–throwing	12.77 (4.45)	13.22 (4.46)	11.58 (5.83)	14.15 (4.84)
Curl‐up	44.81 (23.80)	40.42 (21.71)	38.37 (22.08)	40.15 (21.21)
Push‐up	19.85 (11.85)	19.48 (12.89)	19.25 (13.00)	22.08 (13.27)
*Girls*				
Body mass index (kg/m^2^)	18.75 (2.96)	18.87 (2.82)	18.32 (2.31)	18.77 (2.38)
20‐m shuttle run test	35.28 (16.32)	38.89 (16.78)	32.90 (14.49)	41.90 (14.77)
5‐leaps	7.89 (1.00)	8.17 (0.98)	8.06 (0.85)	8.37 (0.91)
Catching–throwing	10.54 (4.64)	12.61 (4.93)	10.96 (4.82)	13.17 (4.67)
Curl‐up	45.91 (22.95)	44.44 (20.27)	36.24 (21.27)	44.22 (21.71)
Push‐up	28.10 (13.43)	26.93 (12.92)	22.14 (10.78)	29.83 (14.45)

### 3.2 Intervention effects

**FIGURE 1:**
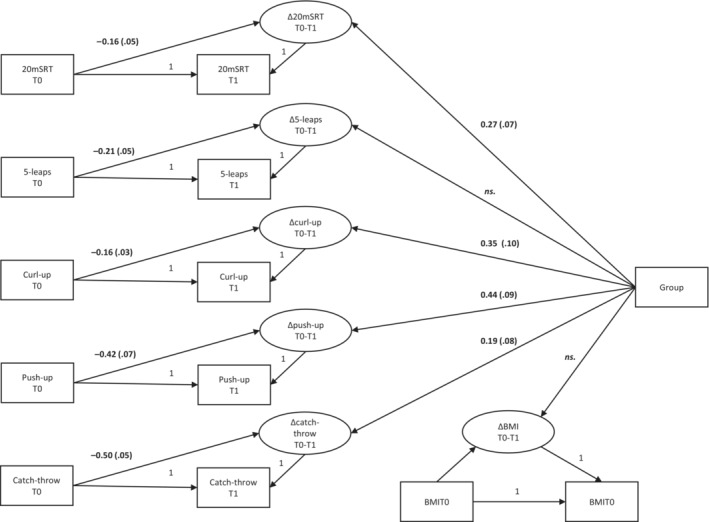
The latent change score model. All paths are significant at the *p* < 0.05 level.

A latent change score model was established to study the effect of the intervention on the change between T0 and T1. All five fitness and motor competence test variables, that is, 20‐meter shuttle run, 5‐leaps, curl‐up, push‐up, and throwing–catching combination tests, along with the body mass index, were used in the final model. The baseline scores were controlled in the analysis. The model (Figure 1) demonstrated a good fit to the data [χ^2^(6) = 8.54, *p* < 0.201, CFI = 0.999, TLI = 0.993, RMSEA = 0.036, 90% CI [0.000, 0.087], SRMR = 0.02]. Model parameters, including regression and correlation estimates, are presented in Table 2. Results revealed that the group condition had a statistically significant positive effect on the latent change in 20‐meter shuttle run (*β* = 0.269, *p* = 0.000, 95% CI [0.141, 0.397]; adjusted mean difference = 5.0 laps), curl‐up (*β* = 0.353, *p* = 0.001, 95% CI [0.154, 0.552]; adjusted mean difference = 7.8 repetitions), push‐up (*β* = 0.442, *p* = 0.000, 95% CI [0.267, 0.617]; adjusted mean difference = 6.5 repetitions) and throwing–catching combination tests (*β* = 0.195, *p* = 0.019, 95% CI [0.033, 0.356]; adjusted mean difference = 1.1 repetitions), but not on 5‐leaps (*β* = 0.060, *p* = 0.402, 95% CI [−0.080, 0.199]; adjusted mean difference = 0.01 m) or body mass index (*β* = −0.066, *p* = 0.580, 95%CI [−0.302, 0.169]; adjusted mean difference = 0.18 kg/m^2^). Based on the two‐group test [χ^2^(6) = 43.63, *p* < 0.001], there was an overall intervention effect, indicating that students in the intervention group developed significantly better than students in the control group when considering all motor competence and HRF variables simultaneously. The effect of gender was also investigated, and based on the Wald test results, the change between pre‐ and posttests were similar between boys and girls in both control and intervention groups (χ^2^(5) = 8.62, *p* = 0.125).

**TABLE 2:** Regression and correlation estimates of the latent change score model.

	Δ20mSRT	Δ5‐leaps	ΔCurl‐ up	ΔPush‐up	ΔCatch–throw	ΔBMI	Group condition
	*β* (SE)	*β* (SE)	*β* (SE)	*β* (SE)	*β* (SE)	*β* (SE)	0 = control
							1 = intervention
20mSRT T0	−0.164 (0.05)**	0.051 (0.05)	0.036 (0.02)	0.027 (0.05)	0.027 (0.04)	−0.009 (0.01)	0.083 (0.23)
5‐leaps T0	0.075 (0.05)	−0.212 (0.05)**	−0.029 (0.02)	0.047 (0.04)	0.028 (0.04)	−0.029 (0.02)	0.256 (0.12)*
Curl‐up T0	0.396 (0.07)**	−0.116 (0.07)	−0.155 (0.03)**	−0.071 (0.06)	−0.016 (0.06)	0.005 (0.02)	0.139 (0.49)
Push‐up T0	0.326 (0.06)**	−0.029 (0.07)	0.098 (0.03)**	−0.418 (0.07)**	−0.012 (0.05)	0.000 (0.02)	−0.095 (0.18)
Catch–throw T0	0.139 (0.07)	0.137 (0.05)*	0.039 (0.02)	0.084 (0.05)	−0.500 (0.05)**	0.015 (0.02)	−0.033 (0.12)
BMI T0	0.139 (0.07)	0.002 (0.08)	0.041 (0.04)	0.026 (0.09)	0.147 (0.07)*	0.020 (0.03)	−0.182 (0.29)
Δ20mSRT							0.269 (0.07)**
Δ5‐leaps	0.083 (0.03)*						0.059 (0.07)
ΔCurl‐up	0.019 (0.03)	0.053 (0.04)					0.353 (0.10)**
ΔPush‐up	0.100 (0.02)**	0.094 (0.03)*	0.127 (0.05)*				0.442 (0.09)**
ΔCatch–throw	0.044 (0.03)	0.077 (0.04)	0.090 (0.04)*	0.079 (0.03)*			0.194 (0.08)*
ΔBMI	−0.074 (0.03)*	−0.130 (0.06)*	−0.007 (0.05)	−0.080 (0.05)	−0.114 (0.05)*		−0.057 (0.12)

*p〈0.05; **p〈0.001.

## 4 DISCUSSION

The aim of our study was to investigate the efficacy of a multicomponent school‐based intervention program on students' motor competence and HRF. In general, we found that students allocated to the intervention group showed significant improvements in most indicators of motor competence and HRF. More specifically, students in the intervention group developed significantly better in 20‐meter shuttle run test, push‐up, curl‐up, and throwing–catching combination tests than students in the control group. Furthermore, neither weight status nor sex moderated the intervention effect.

The overall positive findings of the intervention are in line with previous review studies demonstrating that interventions can improve both motor competence and health‐related fitness among children and adolescents.^9^, ^26^, ^27^, ^28^ It is noteworthy, that professionally instructed activities in schools are targeted to all students, including those who are inactive or who perceive themselves as poor movers, and not just for those who are already physically active. Unfortunately, it is not uncommon for school‐based physical activity interventions to be ineffective among those who need it the most.^55^ Moreover, Hartwig et al. showed based on a pooled analysis from 20 trials that students with lower levels of baseline physical activity benefitted less from school‐based physical activity interventions.^28^ This finding, highlighting organized and guided activities, is especially important for decision‐makers who decide what kind of elements are included in future physical activity programs. Recent analysis of systematic reviews indicated that school‐based interventions have shown limited effects on physical activity,^10^ whereas our study suggests that they may improve motor competence and health‐related fitness.

One key finding of the 5‐month‐long study was the positive development of CRF in intervention group students (+8,4 laps) compared to the control group students (+3,4 laps). In the intervention program, the time allocated to enhancing the activity of students was relatively short, roughly an hour per week, and only a part of the activities specifically developed CRF. Hence, positive development in CRF achieved with relatively low effort, is especially important as researchers and societies have been increasingly worried in declining trends of youth cardiorespiratory fitness levels.^56^ Our finding is mostly in line with, or succeeding, the results of previous intervention programs that have analyzed the effects of vigorous, high‐intensity PA programs on the development of CRF. For example, Lubans et al. reported similar development (+4.1 laps) on 20‐meter shuttle run test at 6‐months follow‐up for adolescent students.^57^ However, Wassenaar et al. found that vigorous PA intervention did not improve students' CRF following a 10‐months long program,^58^ and Martínez‐Vizcaíno et al. reported positive development only for girls (+3,4 laps), but not for boys in a academic‐year‐long high‐intensity PA intervention.^59^ It is clear that positive development of youths' CRF is called for as it has been linked with overall health.^6^, ^7^, ^8^, ^60^

Another key finding of the study was that muscular fitness of the intervention group students developed significantly better compared to the control group. Results in both push‐up (difference = 6,5 repetitions) and curl‐up tests (difference = 7,8 repetitions) improved more in the intervention group compared to the control group which was expected as the performed exercises and tasks in the program systematically and progressively developed muscular fitness attributes. Compared to similar studies, the effect in muscular fitness was substantial. For example, in a previous cluster‐randomized controlled trial among adolescents push‐up test results improved by 2.0 repetitions at 6‐months follow‐up.^61^ In general, the findings are in line with previous studies as shown by a recent review of school‐based interventions targeting muscular fitness.^27^ In the control group, push‐up test results declined from baseline to posttest. A corresponding declining trend in push‐ups has been documented among similarly aged Finnish students in a nationally representative sample.^40^ Taking these together, it is encouraging that a relatively short and easily executable intervention was able to improve students' upper‐body strength and endurance, as well as their abdominal strength and endurance. This positive finding is further amplified by the fact that muscular fitness has been associated with the overall health status of youth.^7^, ^8^, ^24^, ^25^

Intervention effects on motor competence measures were mixed as students in the intervention group developed significantly better in throwing–catching combination test (difference = 1,1) but no differences were found in 5‐leaps. As previously described, the throwing–catching combination test measures students' object control skill proficiency. The increase in object control skills is an important positive finding as object control skills have been shown to be clear predictors of adolescent PA engagement.^12^ It is also notable that both girls' and boys' object control skills developed positively in the current study, which is especially important as previous findings have indicated girls performing more poorly in object control skills than boys.^14^ There might be several reasons for the lack of intervention effect in 5‐leaps test. As a performance, it requires both physical and skill‐related qualities, especially explosive strength, dynamic balance, and rhythmical skills.^62^, ^63^ These multiple requirements might make it more difficult for students to develop in the leaping distance. Moreover, it could also be that the intervention activities were not specific enough for this kind of multifaceted leaping performance to develop, even though the program consisted of several activities that were aimed to enhance locomotor skills. In future studies and intervention programs, it might be reasonable to increase the specificity of the guiding, especially in skill‐related activities.

Equitable access of all students in PA promotion programs should be driven not only because of the clear health benefits but also because improving students' physical performance and their physical activity engagement might help their academic achievement.^64^ Hence, it should be in schools' interest to promote physical activity and fitness programs, especially when program goals are corroborating wider curricular aims. Nevertheless, the feasibility, scalability, and effectiveness of school‐based physical fitness and motor competence interventions are important aspects to consider.^33^, ^34^ All activities and tasks included in the current intervention were designed to be easy to perform by students and easy to be instructed by practitioners. In addition, no additional equipment or special sport venues were needed. Therefore, the intervention can be widely adapted to different schools, and the program activities can be implemented by regular school staff members such as general classroom teachers. From a cost perspective, the PE and classroom activity break components were delivered during regular school hours, and therefore require no additional staff or funding. The only component in this intervention that would require extra funding, is the guided recess activity. Yet, some schools in Finland currently use teaching assistants or older students as recess activators.^65^ These recess activators, with the help of the structured program, might be able to increase students' physical activity but also their physical performance during school hours.

This study includes a number of strengths and weaknesses that should be noted. One of the major strengths of the study is the feasible and scalable program design that enabled it to be integrated into the school day. This also allows the inclusion of all students to the activities without anyone being left out based on gender, ethnicity, fitness status, or motor skill status. In addition, the study utilized a latent change score modeling approach that has been described as a flexible and powerful tool for intervention study analysis.^50^

The limitations of this study should be considered while interpreting the results. The first limitation is our failure to include long‐term follow‐up. As such, we do not know if our intervention effects were sustained over time. Also, the non‐random allocation of schools to different study conditions limits the representativeness of the results. In addition, the measurement of MC was based on two tests whereas a more comprehensive battery would have provided additional information. For example, the MC measures in this study did not include a specific stability component. Also, the measures were product‐oriented meaning that the qualitative aspects of motor skills (process‐oriented measures) were not considered while interpreting the results.

In conclusion, the 5‐month‐long school‐based intervention program was found to be effective in increasing students' Motor competence and health‐related fitness. More specifically, students allocated to the intervention group developed significantly better in 20‐meter shuttle run test, push‐up, curl‐up, and throwing–catching combination tests than students in the control group. This indicates that the intervention program appeared to be effective in increasing students' CRF, muscular fitness, and object control skills. The intervention effect was found to be similar for boys and girls. Findings demonstrate that guided school‐based physical activity programs can be influential in promoting physical fitness and motor competence among early adolescent students.

### 4.1 Perspectives

Negative trends have been documented for early adolescents' motor competence^2^, ^41^ and HRF.^3^, ^4^, ^66^ This is especially worrying from a public health perspective, as weak physical performance in adolescence has been negatively linked to many health outcomes.^5^, ^6^, ^7^, ^8^ School has been clearly identified as an important context to promote motor competence and HRF.^9^, ^10^, ^11^, ^67^ Also recently, an expert panel recognized scalable school‐based interventions as one of the top priorities in physical fitness research.^32^ Our present study described a 5‐month‐long, easy‐to‐administer, cost‐effective intervention program that was conducted during PE lessons, academic lessons, and recess time in Finnish elementary school setting. The results showed that students allocated to the intervention group developed significantly better in different physical performance elements compared to the control group students. This study provides preliminary evidence of the effectiveness of the program; however, future studies are needed with larger and fully randomized samples to confirm the findings.

## FUNDING INFORMATION

This work was supported by the Finnish Ministry of Education and Culture. In addition, MH was funded by the Jenny and Antti Wihuri Foundation and the Finnish Sport Foundation. DRL is funded by an NHMRC Senior Research Fellowship (APP1154507).

## CONFLICT OF INTEREST STATEMENT

The authors declare no conflict of interest.

## SPECIALTY SECTION

Section III: Health, Disease & Physical Activity.

## Supporting information


Supplemental Table 1.

Supplemental Table 2.

## Data Availability

Data are not publicly available due to privacy or ethical restrictions. Contact the corresponding author for more information.
