# Supplementary material for: A scalable school‐based intervention to increase early adolescents' motor competence and health‐related fitness
Source: Scand J Med Sci Sports. 2023 May 25;33(10):2046–57. doi: 10.1111/sms.14410 (PMC10946856; doi:10.1111/sms.14410)
Supplement: Supplementary file 1 — Supplemental Table 1. [file SMS-33-2046-s001.docx]

Supplemental Table 2. Correlation coefficients of the observed variables for control and intervention groups.

|  | **1.** | **2.** | **3.** | **4.** | **5.** | **6.** | **7.** | **8.** | **9.** | **10.** | **11.** | **12.** |
| --- | --- | --- | --- | --- | --- | --- | --- | --- | --- | --- | --- | --- |
| **1. T0 BMI** | 1 | -.27** | -.22** | .01 | -.141 | .07 | .92** | -.22* | -.27 | -.03 | -.17 | -.01 |
| **2. T0 20mSRT** | -.27** | 1 | .67** | .42** | .50** | .57** | -.21* | .89** | .61** | .46** | .52** | .58** |
| **3. T0 5-leaps** | -.26** | .58** | 1 | .52** | .48** | .43** | -.14 | .67** | .82** | .43** | .45** | .55** |
| **4. T0 curl-up** | -.04 | .36** | .37** | 1 | .50** | .32** | .07 | .44** | .39** | .57** | .39** | .31** |
| **5. T0 push-up** | -.28** | .53** | .49** | .45** | 1 | .15 | -.15 | .49** | .47** | .37** | .72** | .31** |
| **6. T0 C-T** | -.03 | .47** | .53** | .33** | .40** | 1 | .19* | .49** | .31** | .28** | .18* | .67** |
| **7. T1 BMI** | .94** | -.25** | -.20* | -.06 | -.27** | .05 | 1 | -.23** | -.30** | -.07 | -.24** | -.05 |
| **8. T1 20mSRT** | -.22* | .86** | .52** | .34** | .49** | .48** | -.22* | 1 | .67** | .47** | .55** | .54** |
| **9. T1 5-leaps** | -.20* | .56** | .85** | .34** | .45** | .49** | -.21* | .54** | 1 | .38** | .55** | .59** |
| **10. T1 curl-up** | -.05 | .41** | .37** | .59** | .39** | .35** | -.09 | .37** | .45** | 1 | .40** | .36** |
| **11. T1 push-up** | -.17* | .51** | .36** | .30** | .73** | .29** | -.19* | .51** | .43** | .46** | 1 | .42** |
| **12. T1 C-T** | -.00 | .46** | .48** | .22* | .37** | .71** | -.02 | .47** | .47** | .33** | .34** | 1 |

*Note.* Intervention group is presented at the bottom triangle and the control group on the top triangle. T0 = pre-tests, T1 = post-tests, BMI = body-mass-index (kg / m^2^), 20mSRT = 20 meter shuttle run test, C-T = catching-throwing combination test.

** p < .01

* p < .05
