# Supplementary material for: A scalable school‐based intervention to increase early adolescents' motor competence and health‐related fitness
Source: Scand J Med Sci Sports. 2023 May 25;33(10):2046–57. doi: 10.1111/sms.14410 (PMC10946856; doi:10.1111/sms.14410)
Supplement: Supplementary file 2 — Supplemental Table 2. [file SMS-33-2046-s002.docx]

Supplemental Table 1. Two example weeks of the 18-week intervention program.

| Intervention component | Example Week 1 | Example Week 2 |
| --- | --- | --- |
| Extended warm-up before regular PE lesson  1 x 20min activity / week | *“Running and postures -game with a pair and music”* ***(cardio-respiratory fitness, muscular fitness)***  Choose a pair. Run in a large circle, choose opposite running directions with your pair. When the music stops, quickly find your pair, and perform the task teacher just called for (e.g. five squats with your pair riding piggyback). When the music starts, continue running in the other direction than before.  *Catch-and-throws in pairs* ***(object control skills)***  Pairs face each others and start throwing and catching with a tennisball. Students are encouraged to try as many different types of throws and catches as possible (e.g. high and low, with and without a bounce, catching with two and one hand, with left and right hand, varying the distance etc). After a while, students try to repeatedly succeed as many times as possible in all of the different types of throwing and catching. After this, students try the same with the catcher having their eyes closed until the other pair shouts ‘open’ while throwing.  *Muscular fitness exercises in groups of three* ***(muscular fitness)***  A group competition where the groups perform a certain total amount of given exercises (e.g. 50 push-ups). The group of three can decide how many performances each person will conduct. The first group that reaches the given total number gets three points, second group two points, and third one point. Different tasks are presented. | *”Never-ending dodgeball”* ***(object control skills and cardio-respiratory fitness)***  Students are moving in a clearly marked area, with 1-3 balls. Anyone can crab a ball and try to hit any other player. Ball must be thrown from the spot where it is picked up. A successful hit in the torso means that the one who got hit must go outside the area to perform a certain task. After that the player can return to the game. If the player catches the ball, the thrower gets out of the field to perform the given task.  *“Wrestling with a pair”* ***(stability and locomotor skills, muscular strength)***  Different wrestling tasks in small space. Example tasks: a) pairs face-to-face, trying to touch the other players shoulders, toes etc., b) trying to get a hug position, ie. hands around the torso below the armpits, following a lift in the air. |
| Long recess  1 x 20min activity / week | *“Protect the nest”* ***(cardio-respiratory fitness)***  With 2-4 teams, the aim is to protect the treasures in your own nest, and to steal the treasures from other teams’ nests. One player can carry one item at a time. Teacher can vary the task for example by changing the way of movement (e.g., running, with only one-foot, by crawling etc).  *“Leaping game”* ***(muscular fitness, locomotor skills)***  Students start from the same spot or line. One is selected as the leaper. The leaper decides how many leaps should be taken and all the others start with their leaps. After that, the leaper tries to reach and touch other players by taking one leap less than the others. If the leaper can touch someone, he/she will become the new leaper and everyone returns to the starting point. If the leaper can’t touch anyone, he/she shouts “fire is loose”, and everyone try to return to the starting point as quickly as possible. The last one to reach the home becomes the new leaper. | *“Push and pull your pair”* ***(muscular fitness)***  The other pair tries to move the fellow student by pushing from the back, and the other one tries to resist as much as possible. From a mark, the roles change so that the first one tries to pull the other while the other tries to decelerate by holding from the hip. Other movements can be similarly applied, with the idea of using counterforces.  *”Catching the ball in different positions”* ***(object control skills)***  Participants work in pairs. One has a ball, and the other one in a given position with eyes closed. The ball is thrown high in the air, and the cathers name is shouted at the same time. The catcher reacts and tries to catch the ball. Different starting positions are used, for example sitting down, lying down etc. After each performance, pairs change their roles. |
| Activity break during academic lessons  5 x 5min activity / week | *Clock* ***(mobility and flexibility)***  Aim of the task is to use your hands to demonstrate a certain time, e.g. quarter past ten. In addition, hands are kept stretched out, and the next time has to be found by spinning the hands in either clockwise or counterclockwise. Task can also be done in pairs, by mirroring the other students’ time. Moreover, similar task can be performed with other bodyparts, e.g., with knees or toes.  *Math fitness* ***(muscular fitness):***  Teacher writes down a calculation/formula to the board, and students are asked to calculate the result in their heads, and to demonstrate the result by doing a similar amount of certain fitness tasks (e.g, push-ups, curl-ups, squats etc.)  *Linehopping with one foot* ***(stability and locomotor skills):***  Seisoteen pulpetin vieressä. Maassa on näkymätön viiva, jonka yli tulee hypätä yhdellä jalalla 10x / jalka. Harjoitusta voi haastaa kuvittelemalla viivan kohdalle aidan, jolloin hyppy suuntautuu kunnolla ylöspäin. Viiva/aita voi olla vartalon vierellä, edessä tai takana  *The Hugo-game* ***(muscular fitness, cardio-respiratory fitness):***  Students are running in place, and following certain commands or signals from the teacher. Each signal means they perform some kind of activity. For example, if the teacher raises his right hand, students do a squat jump and if the teacher raises his left hand, students do five push-ups.    *Chair jumps* ***(lower body strength)***:  All students sit on their chairs, eyes can be closed or kept open. When the teacher or any student in the class yells “UP”, everyone stands up as fast as possible and performs a jump high as possible. After that they sit back down. Different kind of variation can be used (e.g., by first taking a push-up position and then jumping up etc.) | *Mini-triathlon* ***(muscular fitness, cardiorespiratory fitness)***  Teacher can create a short track or the task can be performed by staying in place. Start by doing free-strokes in the air or lying down, continue by running in place, and end with cycling while sitting in chair. Teacher can create “obstacles”, e.g., a fallen tree that has to be jumped over etc.  *Push-ups* ***(muscular fitness)***  Different kind of push-ups using walls, chairs and the floor with pairs. The aim is to perform push-ups in turns. The one who starts does one repetition and the other one continues by doing two and so on.  *Long stretching* ***(mobility and flexibility)***  Three stretching tasks are performed, 20 seconds each, three rounds. Tasks are slavic squat, touching toes while standing with straight legs and side bend.  *Short stretching* ***(mobility and flexibility)***  A set of short stretch-relax exercises are performed in class for lower body and back. For example, hamstring stretching using a chair in five seconds stretch-relax cycles.  *Planking* ***(muscular strength and endurance)***  Students perform a planking set with toes or knees on the ground for example 3 x 40 seconds, with 30 seconds of resting time in between. |
